# Characterization of the Urinary Metagenome and Virome in Healthy Children

**DOI:** 10.3390/biomedicines10102412

**Published:** 2022-09-27

**Authors:** Eman Wehedy, Selvasankar Murugesan, Chinnu Reeba George, Ibrahim F. Shatat, Souhaila Al Khodor

**Affiliations:** 1 College of Health and Life Sciences, Hamad Bin Khalifa University, Doha 34110, Qatar; 2 Research Department, Sidra Medicine, Doha 26999, Qatar; 3 Nephrology Department, Sidra Medicine, Doha 26999, Qatar

**Keywords:** urine metagenome, urine mycobiome, urine virome, healthy children, pediatric population

## Abstract

Recent advances in next-generation sequencing and metagenomic studies have provided insights into the microbial profile of different body sites. However, research on the microbial composition of urine is limited, particularly in children. The goal of this study was to optimize and develop reproducible metagenome and virome protocols using a small volume of urine samples collected from healthy children. We collected midstream urine specimens from 40 healthy children. Using the metagenomics shotgun approach, we tested various protocols. Different microbial roots such as Archaea, Bacteria, Eukaryota, and Viruses were successfully identified using our optimized urine protocol. Our data reflected much variation in the microbial fingerprints of children. Girls had significantly higher levels of Firmicutes, whereas boys had significantly higher levels of Actinobacteria. The genus *Anaerococcus* dominated the urinary bacteriome of healthy girls, with a significant increase in *Anaerococcus prevotii*, *Anaerococcus vaginalis*, and *Veillonella parvula* (*p*-value < 0.001) when compared with that of boys. An increased relative abundance of *Xylanimonas* and *Arthrobacter*, with a significantly high abundance of *Arthrobacter* sp. *FB24* (*p*-value 0.0028) and *Arthrobacter aurescences* (*p*-value 0.015), was observed in boys. The urinary mycobiome showed a significant rise in the genus *Malassezia* and *Malassezia globose* fungus (*p*-value 0.009) in girls, whereas genus *Saccharomyces* (*p*-value 0.009) was significantly high in boys. The beta diversity of the urinary mycobiome was found to differ between different age groups. Boys had significantly more *Mastadenovirus* and *Human mastadenovirus-A* in their urinary virome than girls. With increasing age, we noticed an increase in the relative abundance of the order Caudovirales. Our optimized protocols allowed us to identify the unique microbes for each sex by using an adequate volume of urine (3–10 mL) to screen for the bacteriome, mycobiome, and virome profiles in the urine of healthy children. To the best of our knowledge, this is the first study to characterize the metagenomics profiles of urine in a healthy pediatric population.

## 1. Introduction

The human microbiome is composed of all of the genes of the bacteria, fungi, viruses, archaea, and other types of microbes living inside or on our body [1]. The majority of the human microbiome is located in the gut [2]. The number of gut microbiota is estimated to be more than 10^14^, and the genomic content of the microbiota is 100 times more than that of the human genome [3]. Due to the large populations of bacteria present at this site and the ease with which feces can be obtained as a representative sample for the gut microbiota, the gut microbiome has been extensively studied [4].

In the past, urine was considered a sterile fluid that only became unsterile after infection [5,6,7]. As a result, urine microorganisms were only detected in clinical microbiology laboratories using standard urine culture methods. A few microorganisms can be detected, mostly aerobic and fast-growing bacteria such as *Escherichia coli* and *Klebsiella pneumonia* [8,9]. Unfortunately, these techniques failed to detect anaerobic microbes with slow growth or bacteria with specific nutritional requirements [10,11]. With the development of next-generation sequencing techniques, studies have been conducted on the urinary microbiome. They have revealed that, even though the microbial biomass is lower than that of other body sites, human urinary tracts are dominated by different kinds of microbes. The distribution pattern of these microbes affects urinary tract health [5,12]. While most of the studies used 16S rRNA amplicon sequencing to determine the bacterial composition of the urinary microbiome [13], we used shotgun sequencing, which breaks DNA into smaller pieces to give insights into sample structure and also provides a more comprehensive taxonomy of microbes, such as bacteria, fungi, viruses, and archaea [14].

In the healthy urinary bacteriome, most bacterial taxa are fastidious and slow-growing microbes belonging to one of five major phyla: Firmicutes, Bacteroidetes, Actinobacteria, Fusobacteria, and Proteobacteria, with common genera *Lactobacillus*, *Corynebacterium*, *Staphylococcus*, *Prevotella*, and *Streptococcus* [15]. Many studies found a high level of individual variability [16]. Because men and women have different lower urinary tract structures, the female microbiome is expected to differ from the male microbiome [13,17]. The male urinary bacteriome is significantly enriched in the genus *Corynebacterium*, associated with the skin [16]. Female urinary bacteriomes are mainly dominated by *Lactobacillus*, the main component of the vaginal microbiome [18]. The urinary bacteriome can also be altered by infections of the urinary tract, which can lead to antibiotic resistance [6,19].

Since fungi make up a small percentage of symbiotic microbes and most are uncultivable, they have received far less attention than bacteria [20]. *Candida* species are the most common fungal pathogens associated with the urinary tract. *Cryptococcus*, *Aspergillus*, *Histoplasma, Mucoraceae*, *Blastomyces*, and *Coccidioides* are some of the most common invasive fungal species related to the urinary system. [21]. The urinary tract’s fungal microbiome (mycobiome) composition is significantly influenced by the increased use of antineoplastic medications, systemic immunosuppressive agents, and broad-spectrum antibiotics [22]. The mycobiome is thought to interact with the host and other microbiomes and is linked to some pathological effects [21]. In children, fungal infections of the urinary tract are mostly asymptomatic [23].

The human virome was primarily studied as part of the metagenomic sequencing data, which resulted in an underestimation of the entire virome, particularly RNA viruses [24]. The human virome is composed of bacteriophages, endogenous retroviruses, and eukaryotic viruses [25]. Bacteriophages, which infect bacteria, are the most common viruses and are thought to play a protective role in bacterial distribution control [26]. Viruses are usually not associated with urinary tract infections, but human papillomaviruses, the Torque teno virus, the JC virus, and the BK virus are the most common urinary viruses [27]. In children, there is no standard protocol for assessing the urinary virome. Further research is needed to identify the urinary virome profile and to understand its role in health and disease.

Collecting samples from children requires more precautions and is more challenging [28]. In children with kidney disease or urinary tract issues, not having enough urine sample volume is a common problem [29,30]. Studies of the urinary metagenome and the urinary virome in children are scarce. In this study, we identified reproducible protocols for the urinary metagenome used to characterize the bacteriome, virome, and mycobiome profiles in children, using low-volume urine samples and the shotgun sequencing approach in a healthy pediatric population.

## 2. Material and Methods

### 2.1. Study Design

A total of 40 healthy children were enrolled in this study. The study was approved by the Sidra Medicine Institutional Review Board (IRB) (2019-0045/1538663). We ensured that the study was conducted strictly with the updated revision of the “Declaration of Helsinki” and followed the ICH Guidelines for Good Clinical Practice (CPMP/ICH/135/95), July 1996. Healthy children who met our inclusion criteria were recruited from the Sidra Medicine Outpatient Clinic (OPC). Recruitment for the study took place between September 2020 and December 2021. All participants had a physical examination, and parental informed consent/child assent was obtained before inclusion in the study. Children 1–18 years of age who were not suffering from any chronic diseases, had not received antibiotic treatment in the last two months, and had consented to participate in the study were included. Antibiotic therapy in the previous two months, gross anatomical abnormalities, congenital anomalies, and the presence of any chronic disease were all considered exclusion criteria. All data collected from the study subjects, including age, gender, and nationality, were recorded in the Research Electronic Data Capture (REDCap) electronic database using study codes without participant identifiers. Mid-stream urine samples were collected by the participants and their guardians in a sterile urine container. Samples were aliquoted into 3–10 mL aliquots and kept at −20 °C until further processing.

### 2.2. DNA Extraction for Urinary Metagenome

Microbial DNA extraction from urine samples was tested using three different treatments, and the extraction was processed as described by Moustafa et al. in 2018 [31]. As a pilot step, we collected urine samples from 5 healthy donors. Three different treatments were tested as follows: treatment #1 included centrifugation of the urine sample at 3400× *g* for 1 h; treatment #2 included concentration of the urine samples using Amicon Ultra-15 at 3400× *g* for 1 h; and treatment #3 included concentration of the urine samples using Amicon Ultra-15 (3400× *g*, 1 h), with a combination of lysozyme and zymolase enzymes. We decided to proceed with treatment #3 in this study as it showed the expression of all the tested microbial markers. Briefly, a total of 3–10 mL of urine samples were thawed on ice and transferred to an Amicon Ultra-15 (MERCK, Millipore, Cork, Ireland) filter unit with a cut-off 3 KD (Ultracel -3K) to retain all small-sizes microbes, and it was then centrifuged for 1 h at 10 °C at 3400× *g*. The final volume was 230–300 µL. The filter column was later washed with 100 µL of sulfate magnesium (SM) buffer to collect the remaining sample. We mixed 300 µL of lysis buffer (20 mM Tris-Cl, pH 8.0, 2 mM EDTA, and 1.2% Triton X-100) with the sample, and it was vortexed and incubated for 10 min at 75 °C. An enzymatic lysis mix containing 60 µL lysozyme (200 µg/mL), 5 µL zymolase (10 units), and 20 IU RNase I was added to each sample after being cooled to room temperature and incubated at 37 °C for 1 h in the thermomixer (Eppendorf, ThermoMixer, Germany). The samples were later incubated at 55 °C overnight (~18 h) after the addition of 100 µL 10% SDS and 42 µL Proteinase K (20 mg/mL), and the extraction continued as described [31].

### 2.3. RNA Extraction for Urinary Virome

To optimize the viral nucleic acid extraction protocol, the viral nucleic acid was extracted using four different protocols: (i) QIAamp viral RNA mini kit (Qiagen, Germany), (ii) split RNA extraction kit (Lexogen, Austria), (iii) the protocol previously reported by Norman et al. 2015 [32], and (iv) the protocol published by Shokoprov et al. in 2018 [33], using the five donor samples before proceeding with the study samples. In the split RNA extraction kit, we performed the following modifications to the manufacturer’s instructions. Briefly, urine samples (3–10 mL) were thawed on ice. Next, the samples were filtered with a 0.8 µm syringe-mounted filter (MCE membrane, Millipore, Cork, Ireland), followed by filtration twice with a 0.45 µm syringe-mounted filter (SFCA, Thermo Scientific, Rochester, NY, USA). The samples were then transferred to an Amicon Ultr-15 (Ultracel -3K) (MERCK, Millipore, Cork, Ireland) filter unit and centrifuged for 40 min at 4 °C at 3400× *g*. The concentrated urine samples (~200 µL) were collected in a sterile 1.5 mL tube. An equal volume of isolation buffer was mixed with the concentrated urine sample, and the extraction process continued according to the manufacturer’s instructions.

We proceeded with the split RNA extraction kit for the viral total RNA extraction in this study as it produced 60% viral sequence reads of the total number of sequence reads.

### 2.4. cDNA Synthesis and Virus Amplification

The cDNA of the extracted viral nucleic acid from urine was synthesized using Superscript™ IV First-Strand Synthesis System (Invitrogen, Thermo Scientific, Vilnius, Lithuania). Hexamer oligonucleotides and 11 µL of the purified viral-like particles (VLP) were used for the reverse transcription (RT) reaction as described by the manufacturer’s protocol, with a final volume of 20 µL. The RT product was used as a DNA template for viral amplification with the Illustra Genomiphi V2 DNA amplification kit (GE Healthcare, Little Chalfont, Buckinghamshire, UK). Three independent replicates were performed for each sample with a 1 µL cDNA template. After amplification, the products of all replicates (20 µL each) were pooled and mixed with the remaining 17 µL RT products and quantified using a Qubit 4 fluorometer with a Qubit dsDNA HS Assay kit (Invitrogen, Eugene, Oregon, USA) [33].

### 2.5. Library Preparation and Shotgun Sequencing

The microbial DNA was sheared into small fragments using a mechanical method by ultrasonication (Covaris, LE220, Massachusetts, USA). We normalized 100 ng of DNA into 52.5 µL and transferred it to Covaris microtubes. The DNA was sheared using the following setting: Duty Factor 20%, Intensity 5, Peak/Displayed Power 450 W, and cycle/Burst 200, with a fragment size of 350 bp. The libraries were prepared using Nano DNA Library preparation (Illumina, San Diego, CA, USA) according to the manufacturer’s protocol, and each sample was labeled using IDT-ILMN TruSeq DNA UD Indexes (96 Indexes). The library quality was checked by running the samples in an Agilent 2100 bioanalyzer (Agilent Technologies, Waldbronn, Germany) using a high-sensitivity DNA chip (Agilent Technologies, Germany). The libraries were quantified using a Qubit 4 fluorometer (Invitrogen, Thermo Scientific, Singapore), and the concentration was calculated in nM. Next, MiSeq was used to sequence the libraries. A total of 25 nM from each sample was pooled, and the final concentration of the pooled libraries was measured using Qubit 4. The library denaturation and the PhiX control denaturation were done according to the manufacturer’s protocol. The final sample of 600 µL (10 pM) with 5% PhiX control was loaded to the MiSeq (Illumina, San Diego, USA) for sequencing.

### 2.6. Data Analysis and Statistical Analysis

The metagenomics analysis server MG-RAST (https://www.mg-rast.org (accessed on 2 February 2022)) was used to annotate the metagenome structure of the samples [34]. The sequence data were processed as follows: The sequence statistics were calculated using DRISEE and Jellyfish tools [35,36]. The adapters were removed using skewers, and denoising and normalization were done through fastq-mcf [37,38]. The human DNA contamination was removed using the bowtie2 tool [39]. Furthermore, RNA feature identification, RNA clustering, and RNA similarity search were performed using the SortMeRNA, CD-HIT, and Blat tools, respectively. We used the NCBI Refseq database for microbial profiles [40,41,42]. FastViromeExplorer was used to annotate human virome data (https://github.com/saima-tithi/FastViromeExplorer (accessed on 10 February 2022)) [43]. Microbiome profiles and alpha (Observed, Chao1, Shannon, and Simpson) and beta diversities of the urine metagenome and virome were generated using the R-package “MicroEco” [44]. To identify the differentially abundant taxa, an unpaired multiple *t*-test and a Mann–Whitney’s comparison of the ranking test were performed using GraphPad Prism version 9.0.1 [45]. Beta diversity and Bray Curtis distance matrices were calculated and graphically represented through a principal coordinate analysis (PCA) and tested using PERMANOVA. A *p*-value < 0.05 was considered statistically significant.

## 3. Results

### 3.1. Demographic Data

**Figure 1:**
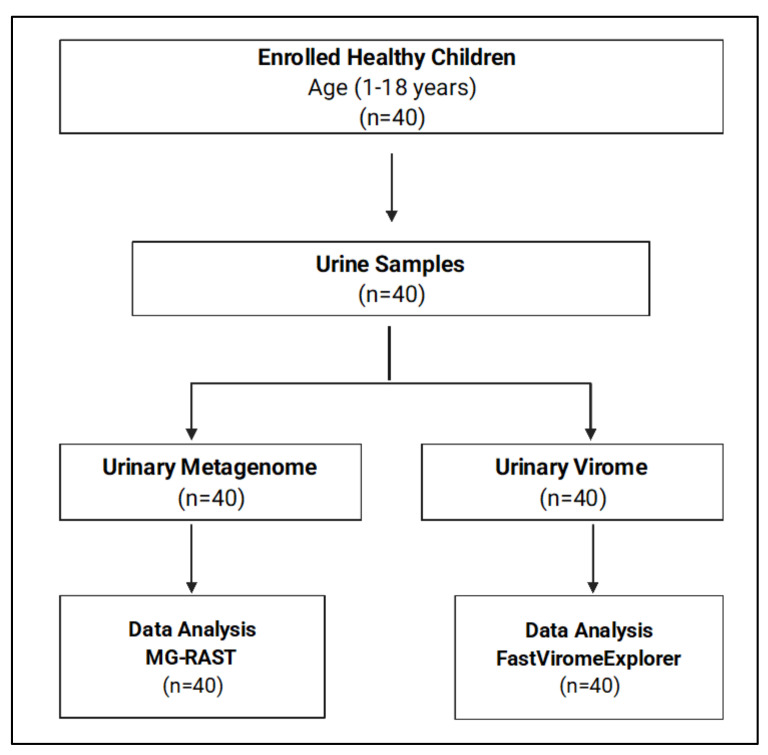
Flowchart of the study participants and sample processing.

Forty healthy children from different nationalities were included in this study. The demographic data are summarized in Table 1. We classified the participants into three age groups: Age 1–5 (*n* = 10), Age 6–10 (*n* = 19), and Age > 10 (*n* = 11). A total of 40 urine samples were collected and sequenced for both urinary metagenome and urinary virome, as shown in Figure 1.

### 3.2. Urinary Metagenome Composition in Healthy Children

We used samples from five healthy donors to test the different protocols. Treatment 3 (T3) showed expressions of all tested bacterial and fungal markers (Figure S1A) when we performed PCR amplification for the extracted DNA from the urine samples (the primers list for the tested genes is summarized in the Supplementary Data (Table S1). Compared with the other two treatments, we found that the intensity of the bands of 16S rRNA, 18S rRNA, and ITS genes were collectively the best in T3. After that, we proceeded with the shotgun sequencing, and the data showed the presence of various microbial communities, including Archaea, Bacteria, Eukaryotes, and Viruses (Figure S1B). For the urinary virome, the Lexogen total RNA extraction kit showed a high percentage of viral reads (up to 60% of the total sequence reads) compared with that of the other protocols (Figure S1C). Approximately 69% of the reads in Norman’s protocol [32] were bacterial, and 28% were Eukaryota. The dominant domain was bacteria in both Shokoprov’s protocol [33] and the Qiagen viral kit protocol (Figure S1C).

**Figure 2:**
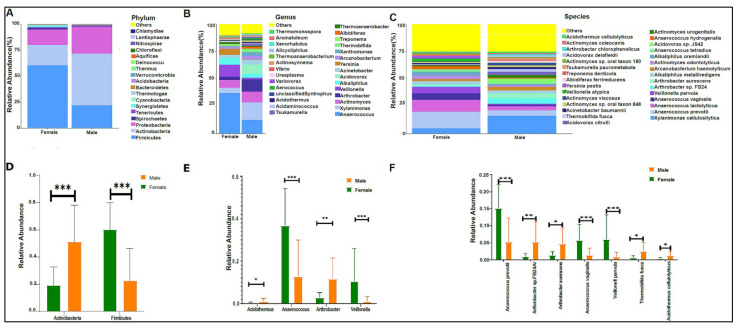
The urinary bacteriome and gender. *Y*-axis shows % of the relative abundance; *X*-axis indicates the bacterial abundance in boys and girls. A different color shows each taxonomic category: (**A**) at the phylum level; (**B**) at the genus level; (**C**) at the species level. Bar graphs of (**D**) differentially abundant bacterial phyla, (**E**) at the genus level, and (**F**) at the species level among the two groups. Girls (green) and boys (orange), * *p* < 0.05, ** *p* < 0.01, and *** *p* < 0.001.

When we applied our modified protocol to the study samples, we found an average of 787,769 sequence reads per sample. Bacteria were the highest abundant domain, followed by Eukaryotes. The urinary bacteriome profiles of boys had a unique composition compared with that of girls at the phylum level (Figure 2A), genus level (Figure 2B), and species level (Figure 2B,C). Firmicutes, Actinobacteria, and proteobacteria were the most abundant phyla in the urinary bacteriome (Figure 2A). Firmicutes was significantly higher in girls compared with boys, with a *p*-value of 0.0001. Conversely, Actinobacteria was significantly higher in boys than in girls, with a *p*-value of 0.00032 (Figure 2D). Girls had significantly higher levels of *Anaerococcus* and *Veillonella* genera than boys did, with *p*-values of 0.0003 and 0.0004, respectively (Figure 2E), and boys had significantly higher *Arthrobacter* and *Acidothermus* than girls did, with *p*-values of 0.0016 and 0.017, respectively (Figure 2E). At the species level, *Anaerococcus vaginalis* (*q*-value 0.00017), *Anaerococcus prevotii* (*p*-value 0.00036), and *Veillonella parvula* (*p*-value 0.00036) were significantly high in female children. In contrast, male children had significantly higher levels of *Arthrobacter sp. FB24, Arthrobacter aurescens*, *Thermobifida fusca*, and *Acidothermus celluloyticus*, with *p*-values of 0.0028, 0.015, 0.014, and 0.017, respectively (Figure 2F).

**Figure 3:**
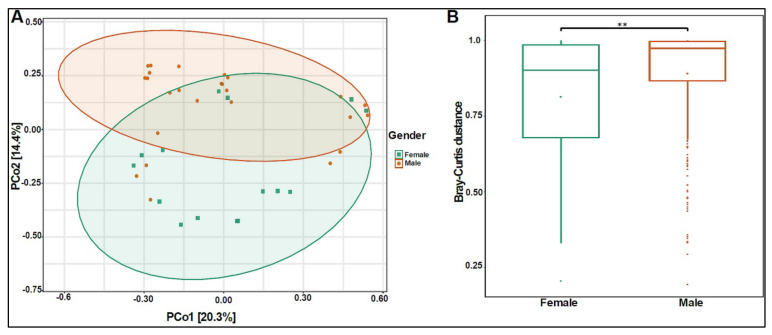
Beta diversity of urinary bacteriome between gender. (**A**) Principal Coordinate Analysis (PCoA) based on Bray–Curtis dissimilarities of urinary bacteriome. Axes are scaled to the amount of variation explained; (**B**) boxplots of Bray–Curtis distance matrix among the two groups. Girls (green) and boys (orange). ** *p* < 0.01.

The beta diversity analysis showed that the bacterial composition was significantly different between boys and girls, with a *p*-value of 0.005 (Figure 3A,B).

**Figure 4:**
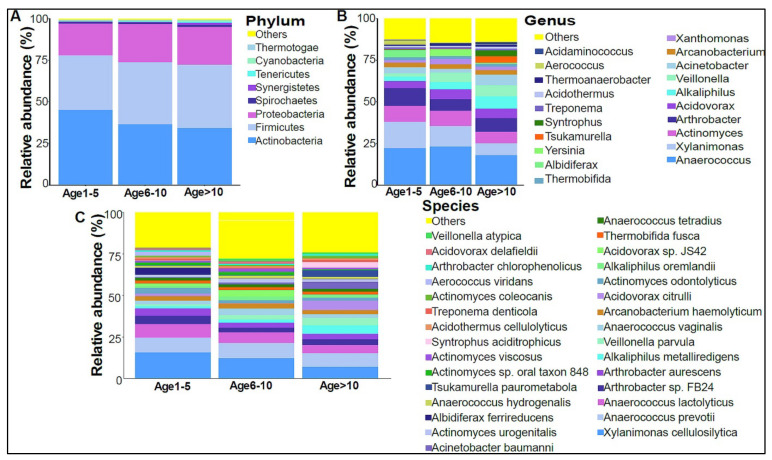
The urinary bacteriome and age groups. *Y*–,axis shows % of the relative abundance; *X*–,axis indicates the bacterial abundance in the three age groups. Each taxonomic category is shown using a different color: (**A**) at the phylum level; (**B**) at the genus level; (**C**) at the species level. Age 1–5 (*n* = 10), Age 5–10 (*n* = 19), and Age > 10 (*n* = 11).

**Figure 5:**
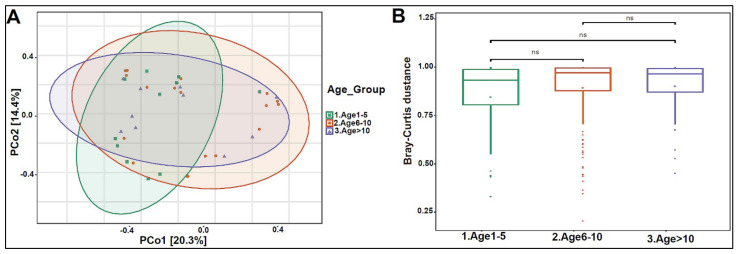
Beta diversity of urinary bacteriome between age groups. (**A**) Principal Coordinate Analysis (PCoA) based on Bray-Curtis dissimilarities of urinary bacteriome. Axes are scaled to the amount of variation explained. (**B**) Boxplots of Bray-Curtis distance matrix among the three groups. Age 1–5 (green), Age 6–10 (orange), and Age > 10 (purple). Not significant (ns).

In addition, we divided the study subjects into three age groups: Age 1 to 5, Age 6 to 10, and Age >10 years old. Actinobacteria levels were slightly higher in the Age 1–5 group, with lower levels in the older age groups (Figure 4A). No statistically significant differences were observed at the genera and species levels (Figure 4B,C). The beta diversity showed that the bacterial composition was not significantly different between the age groups (Figure 5A,B). Furthermore, no statistically significant differences in alpha diversity indices were found between the age groups (data not shown). The bacterial composition did not show significant dissimilarity between the eight nationalities, with a *p*-value of 0.19 (Figure S2A).

**Figure 6:**
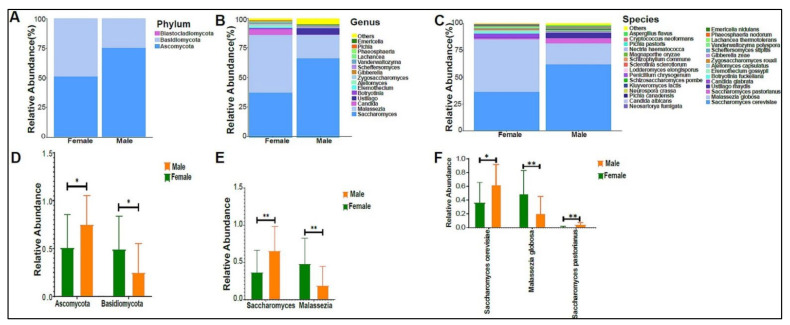
The urinary mycobiome and gender. *Y*-axis shows % of the relative abundance; *X*-axis indicates the fungal abundance in males and females. Each taxonomic category is demonstrated using a different color: (**A**) at the phylum level; (**B**) at the genus level; (**C**) at the species level. Bar graphs of (**D**) differentially abundant fungal phyla, (**E**) at the genus level, and (**F**) at the species level among the two groups. Girls (green) and boys (orange), * *p* < 0.05 and ** *p* < 0.01.

**Figure 7:**
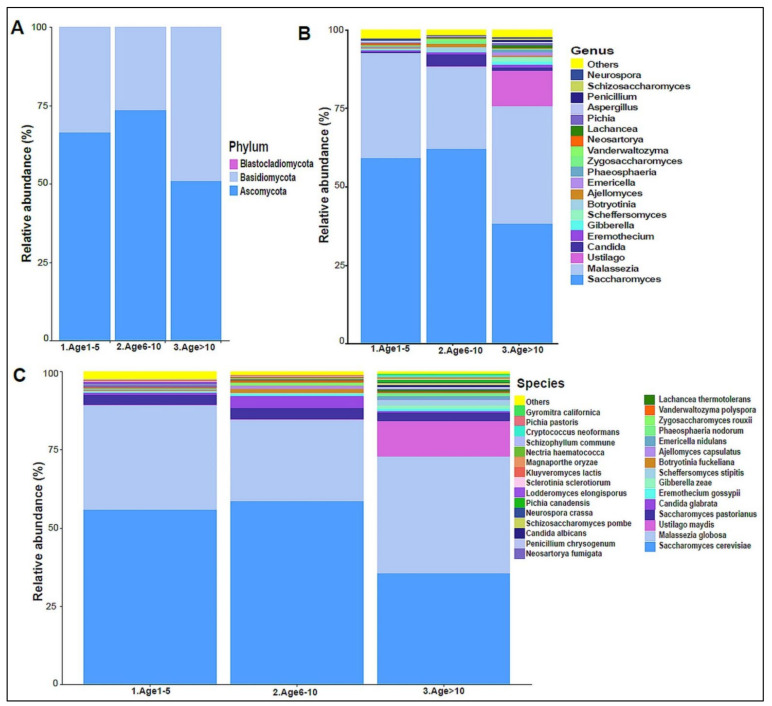
The urinary mycobiome and age groups. *Y*-axis shows % of relative abundance; *X*-axis indicates the fungal abundance in the three age groups. A different color shows each taxonomic category: (**A**) at the phylum level, (**B**) at the genus level, (**C**) at the species level. Age 1–5 (*n* = 10), Age 5–10 (*n* = 19), and Age > 10 (*n* = 11).

**Figure 8:**
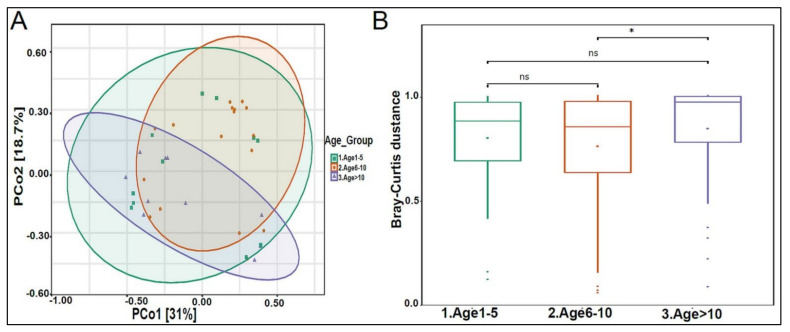
Beta diversity of urinary mycobiome between age groups. (**A**) Principal Coordinate Analysis (PCoA) based on Bray-Curtis dissimilarities of urinary mycobiome. Axes are scaled to the amount of variation explained; (**B**) boxplots of Bray-Curtis distance matrix among the three groups. Age 1–5 (green), Age 6–10 (orange), and Age > 10 (purple). Not significant (ns). * *p* < 0.05.

The urinary mycobiome was dominated by the phyla Ascomycota and Basidiomycota (Figure 6A). The fungal profile composition in male children was different compared with that of female children at the phylum level (Figure 6A), genus level (Figure 6B), and species level (Figure 6C). Boys had significantly higher levels of Ascomycota than girls did (*p*-value 0.044), and girls had significantly higher levels of Basidiomycota (*p*-value 0.044) (Figure 6D). *Saccharomyces pastorianus* (*p*-value 0.001) and *Saccharomyces cerevisiae* (*p*-value 0.017) were significantly higher in male children, while *Malassezia globose* (*p*-value 0.009) was higher in female children (Figure 6E,F). The beta diversity did not significantly differ between boys and girls (data not shown). The profiling of the three age groups’ urinary mycobiome showed no statistical significance (Figure 7A–C). The beta diversity was statistically significant between the Age 6–10 and Age > 10 groups, with a *p*-value of 0.017 (Figure 8A,B). The beta diversity of the urinary mycobiome did not show a statistically significant difference between the eight nationalities, with a *p*-value 0.371 (Figure S2B).

### 3.3. Urinary Virome Profile of Healthy Children

**Figure 9:**
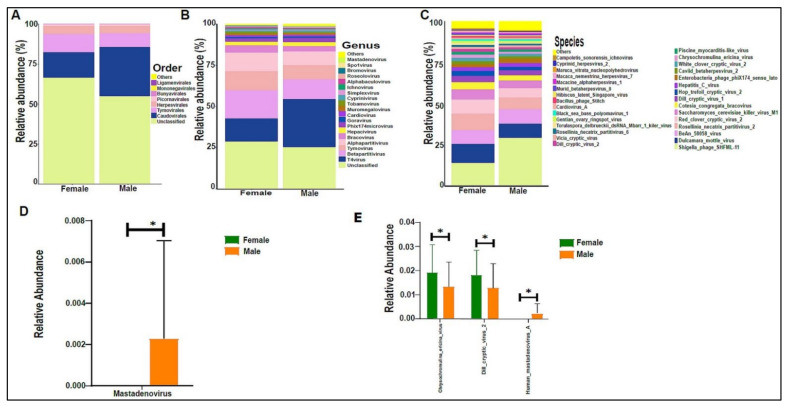
The urinary virome and gender. *Y*-axis shows % of the relative abundance; *X*-axis indicates the viral abundance in boys and girls. Each taxonomic category is demonstrated using a different color: (**A**) at the phylum level; (**B**) at the genus level; (**C**) at the species level. Bar graphs of (**D**) differentially abundant at the genus level and (**E**) at the species level between the two groups. Girls (green) and boys (orange), * *p* < 0.05.

**Figure 10:**
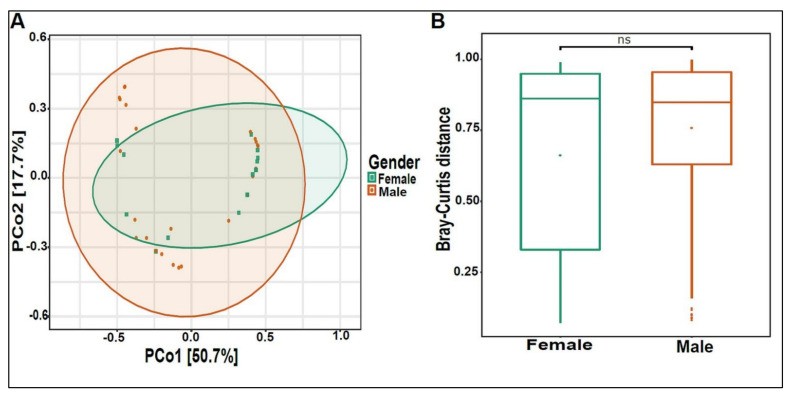
Beta diversity of urinary virome between genders. (**A**) Principal Coordinate Analysis (PCoA) based on Bray-Curtis dissimilarities of urinary virome. Axes are scaled to the amount of variation explained. (**B**) Boxplots of Bray-Curtis distance matrix between the two groups. Girls (green) and boys (orange). Not significant (ns).

**Figure 11:**
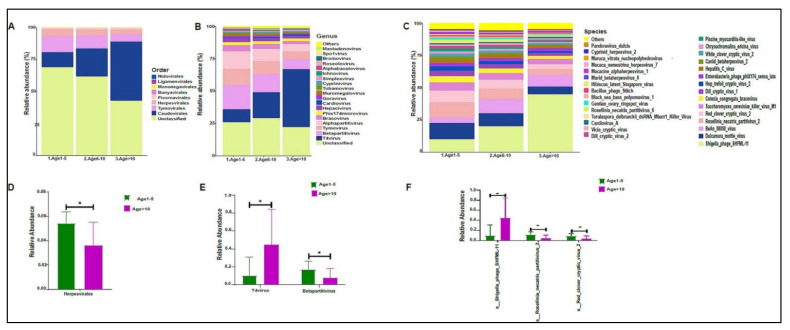
The urinary virome and age groups. *Y*-axis shows % of the relative abundance; *X*-axis indicates the viral abundance in the three age groups. A different color shows each taxonomic category: (**A**) at the phylum level; (**B**) at the genus level; (**C**) at the species level. Bar graphs of (**D**) differentially abundant at the viral order, (**E**) at the genus level, and (**F**) at the species level among the age groups. Age 1–5 (green) and Age > 10 (purple), * *p* < 0.05. Age 1–5 (*n* = 10), Age 5–10 (*n* = 19), and Age > 10 (*n* = 11).

**Figure 12:**
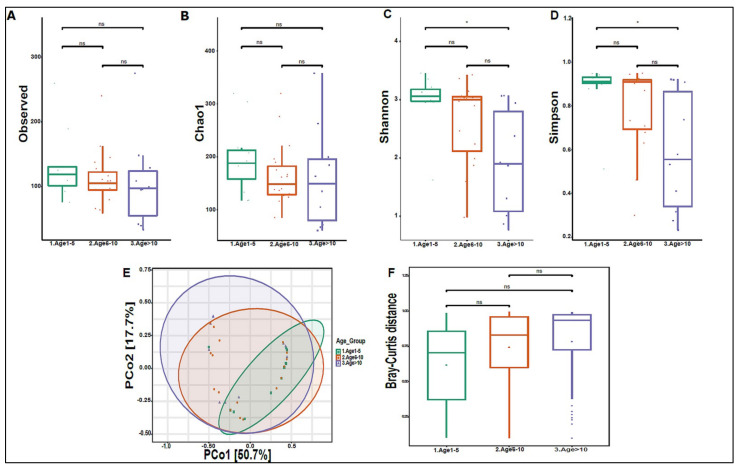
Diversity of urinary virome between age groups. Alpha diversity measures for the three age groups. Species richness was (**A**) measured by the number of OTUs observed or (**B**) estimated by the Chao1. Alpha diversity measured (**C**) the Shannon index and (**D**) the Simpson index. (**E**) Beta diversity: Principal Coordinate Analysis (PCoA) based on Bray-Curtis dissimilarities of urinary virome; axes are scaled to the amount of variation explained. (**F**) Boxplots of Bray-Curtis distance matrix among the age groups. Age 1–5 (green), Age 6–10 (orange), and Age > 10 (purple). Not significant (ns). * *p* < 0.05.

On average, we obtained 4,443,948 viral sequence reads per sample. The urinary virome of the healthy children showed Caudovirales, Tymovirales, and Herpesvirales to be the most abundant orders (Figure 9A). *T4Virus*, *Betapartitivirus*, *Tymovirus*, and *alphapartitivirus* were the most abundant viral genera (Figure 9B). At the species level, *Shigella phage SHFML-11* was the most abundant virus (Figure 9C). Of note, the genus *Mastadenovirus* and the *Human Mastadenovirus-A* virus were statistically significant in male children compared with female children, with a *p*-value of 0.035 (Figure 9D,E). *Dill cryptic virus 2* and *Chrysochromulina ericina virus* were higher in girls than in boys, with *p*-values of 0.028 and 0.033, respectively (Figure 9E). The beta diversity revealed no significant differences between boys and girls (Figure 10A,B). We noticed an increase in the abundance of order Caudovirales, genus *T4Virus*, and *Shigella phage SHFML-11* with age (Figure 11A–C). The Age 1–5 group had a significantly higher abundance of *Herpesvirales* (Figure 11D), *Shigella phage SHFML-11, Rosellinia necatrix partitivirus 2*, and *red clover cryptic virus 2* compared with the Age > 10 group (Figure 11F). The Shannon and Simpson indices showed a significant decrease in the Age > 10 group compared with the Age 1–5 group, with *p*-values of 0.015 and 0.022, respectively (Figure 12C,D). However, the beta diversity was not statistically significant between the three age groups (Figure 12E,F). In addition, we did not observe any statistically significant difference in beta diversity between the eight nationalities, with a *p*-value of 0.146 (Figure S2C)).

## 4. Discussion

Diverse microbes reside in the urinary tract, including normal and pathogenic (causing UTIs) microbes [46]. Some UTIs are eventually asymptomatic, while others become recurrent, and in some cases, the infection becomes chronic and difficult to treat [47]. We believe this is the first study to investigate three biomes—the bacteriome, mycobiome, and virome components in healthy children’s urine. We performed metagenomic shotgun sequencing for 40 urine samples. Each sample was sequenced twice, once for the complete urinary metagenome profile and also for the urinary virome. The data showed that the urinary metagenome had an average count of 330 viruses/sample, while the urinary virome had an average count of 35,117 viruses/sample. Since metagenome data cover bacteria, eukaryotes, fungi, and other microbial profiles, there is a meager number of viruses in the urinary metagenome compared with that of the virome protocol. In the virome protocol, we focused exclusively on viruses’ profiles. Since viruses are considered important in pathogenesis and pathophysiology, it is crucial to accurately identify the virome profile in some diseases, such as kidney diseases [48,49].

To ensure complete coverage of all microbes in the samples, we used a concentrator instead of centrifugation to concentrate the urine sample and avoid missing low-weight microbes in the supernatant [48,49]. Furthermore, we used a mixture of lysozyme and zymolase enzymes to extract DNA for metagenomic sequencing; lysozyme has a lysis effect on bacteria cell walls [50], and zymolase exhibits lysis activity on fungi cell walls [51].

Our findings revealed that female children have significantly more Firmicutes bacteria than male children. The urinary microbiome of adult women is similar to the microbial composition of the vaginal microbiome. Still, it differs from the gut microbiota [52], and the ratio of Firmicutes to *Lactobacillus* species has been confirmed as an indicator of vaginal health in adult females [53,54]. Female children had significantly higher abundances of *Anaerococcus vaginalis*, *Anaerococcus prevotii,* and *Veillonella parvula* in their urinary bacteriome profile than male children. Recent studies revealed that adult women have a high level of *Lactobacillus* [55], and girls after puberty have similar urobiome compositions to adult females [56]. This is consistent with our findings that *Lactobacillus* species like *Lactobacillus gasseri*, *Lactobacillus iners*, and *Lactobacillus johnsonii* are more prevalent in female children around the age of ten than in younger females. Male children had a higher abundance of *Arthrobacter* species than female children, even though the urobiome of boys did not show an enrichment of sex-related bacteria [57]. Surprisingly, the urinary bacteriome structure did not change significantly across age groups, supporting the theory that the stability of the human microbiome structure is critical for maintaining good health [58,59,60,61,62].

The core fungal phyla in the urinary mycobiome were Ascomycota and Basidiomycota, with a high abundance of *Saccharomyces* and *Malassezia,* both of which were reported as commensal flora [63]. Female children’s urinary mycobiome had a significantly higher abundance of the *Malassezia* genus and *Malassezia globose* fungus than the urinary mycobiome of male children. *Malassezia* was identified in different body sites, including the skin, breast milk, and the gut, and it is thought to be associated with host immunity [64,65,66]. *Saccharomyces species* are known for their enzymatic and fermenting activities [67,68]. Male children showed a significantly higher abundance of *Saccharomyces pastorianus* and *Saccharomyces* cerevisiae. This finding was not reported before, and the exact role of *Saccharomyces* sp. in the male urinary tract at a healthy state is still unknown.

Our results showed that Caudovirales, Tymovirales, and Herpesvirales are the dominant viruses of the urinary virome. Caudovirales phages have been reported as a dominant component of the gut virome in various pediatric and adult studies, and their abundance has a high sensitivity to inflammatory conditions [25,32,69,70]. These phages are known to modulate the structure of other microbial communities in the gut [71,72]. Tymovirales are viruses that mainly affect plants and could be transmitted to humans via food consumption [73]. The role of these viruses in the urinary tract is unknown. We hypothesized that a Tymovirales colonization in our healthy cohort’s urinary tract was transmitted from the gut to the bladder during the urine filtration process. Herpesviruses are related to various diseases and are pathogenic [74,75]. Nevertheless, recent studies showed that herpesviruses are part of healthy human viromes [76,77]. With increasing age, the Shannon and Simpson diversity indices decreased, while the relative abundance of the Caudovirales gradually increased. Our study showed that the virome structure in the urinary tract is highly diverse, that the core viruses are stable with age, and that dysbiosis, a change in the microbial structure of the virome community, can lead to pathological consequences.

Our study has some limitations, including the small number of participants and larger cohorts needed to validate our findings. Contamination is another concern, as despite our efforts to take all possible precautions during sample collection, such as providing sterile containers with sterile gloves and explaining how to collect midstream urine to participants and their guardians, the samples may still have contained contaminants from the skin or the urinogenital system. To minimize contamination, catheterized specimens or cystoscopy collection of the urine from the urinary bladder are recommended, especially when dysbiosis of the urinary microbiome is expected to play a role in disease pathophysiology. Although urine sample concentration increases the sensitivity of microbiome detection, the results may be biased if certain microorganisms are very abundant at the expense of others.

## 5. Conclusions

In this study, we profiled the urinary microbiome of healthy children. The urinary bacteriome and urinary mycobiome of girls and boys differed significantly. In comparison with that of other body sites, the urinary virome has a unique structure. Except for some changes in the urinary bacteriome of females during puberty, the microbial communities remained relatively stable as they grew older. This finding supports the theory that the microbiome structure is formed at a young age but can change depending on factors such as drug intake, antibiotic treatment, diet, and other environmental factors.

## Figures and Tables

**Table 1:** Demographic data of the study subjects.

Number of Participants	40
**Sex *n* (%)**	
Male	25 (62.5)
Female	15 (37.5)
**Age**	
Average in years (Standard deviation)	8.275 (4.49)
Age 1–5	*n* = 10
Age 6–10	*n* = 19
Age 11–17 (Age > 10)	*n* = 11
**Nationalities *n* (%)**	
Algerian	2 (5)
Egyptian	13 (32.5)
Indian	11 (27.5)
Qatari	1 (2.5)
Spanish	5 (12.5)
Sri Lankan	3 (7.5)
Syrian	2 (5)
British	3 (7.5)

## Data Availability

The urine metagenome raw and processed data are publicly available in the MG-RAST database (Project ID: mgp103481). The urine virome raw and processed data are available in the BioProject database (PRJNA850668). The software, tools, and codes used to generate the data for this manuscript are available in Supplementary source codes and software list.

## Supplementary Materials

The following supporting information can be downloaded at: https://www.mdpi.com/article/10.3390/biomedicines10102412/s1, Figure S1: Optimization of urinary metagenome and virome protocols; Figure S2: Beta diversity of the different nationalities of the healthy children; Table S1: Primers list of bacterial and fungal marker genes; Supplementary source codes and software list: Scripts and bioinformatics workflow.

## Abbreviations

IRB	Institutional review board
OUT	Operational taxonomic unit
REDCap	Research Electronic Data Capture
RT	Reverse transcription
SM	Sulfate magnesium
UTIs	Urinary tract infections
VLP	Viral-like particles
