# Supplementary material for: Characterization of the Urinary Metagenome and Virome in Healthy Children
Source: Biomedicines. 2022 Sep 27;10(10):2412. doi: 10.3390/biomedicines10102412 (PMC9599034; doi:10.3390/biomedicines10102412)
Supplement: Supplementary file 1 [file biomedicines-10-02412-s001.zip › Figure S1.pdf]

A

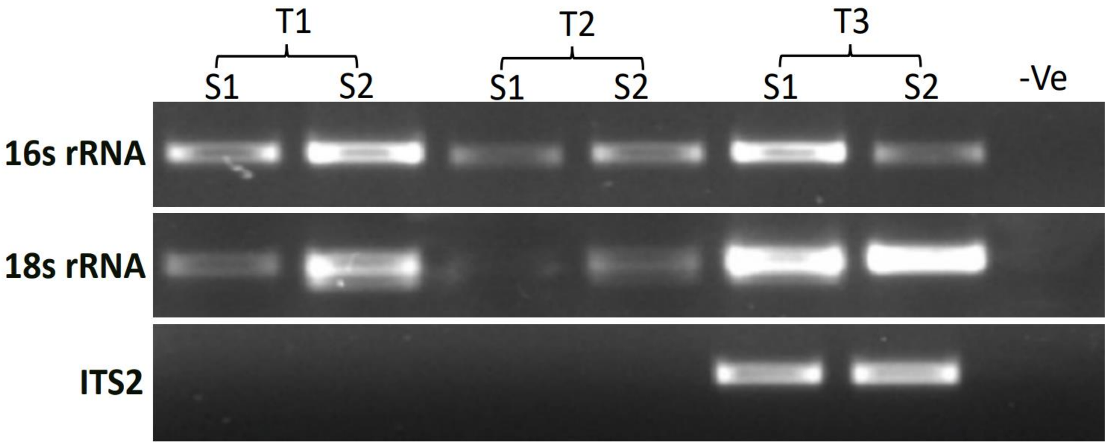
T1
T2
T3
S1
S2
S1
S2
S1
S2
-Ve
16s rRNA
18s rRNA
ITS2

B

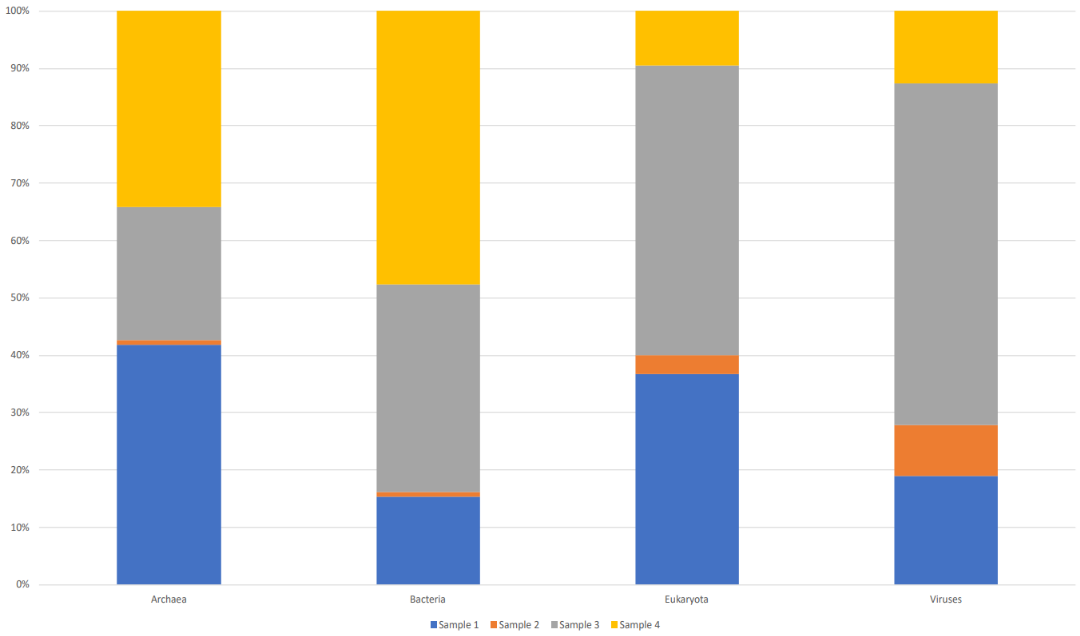
100%
90%
80%
70%
60%
50%
40%
30%
20%
10%
0%
Archaea
Bacteria
Eukaryota
Viruses
Sample 1
Sample 2
Sample 3
Sample 4

C

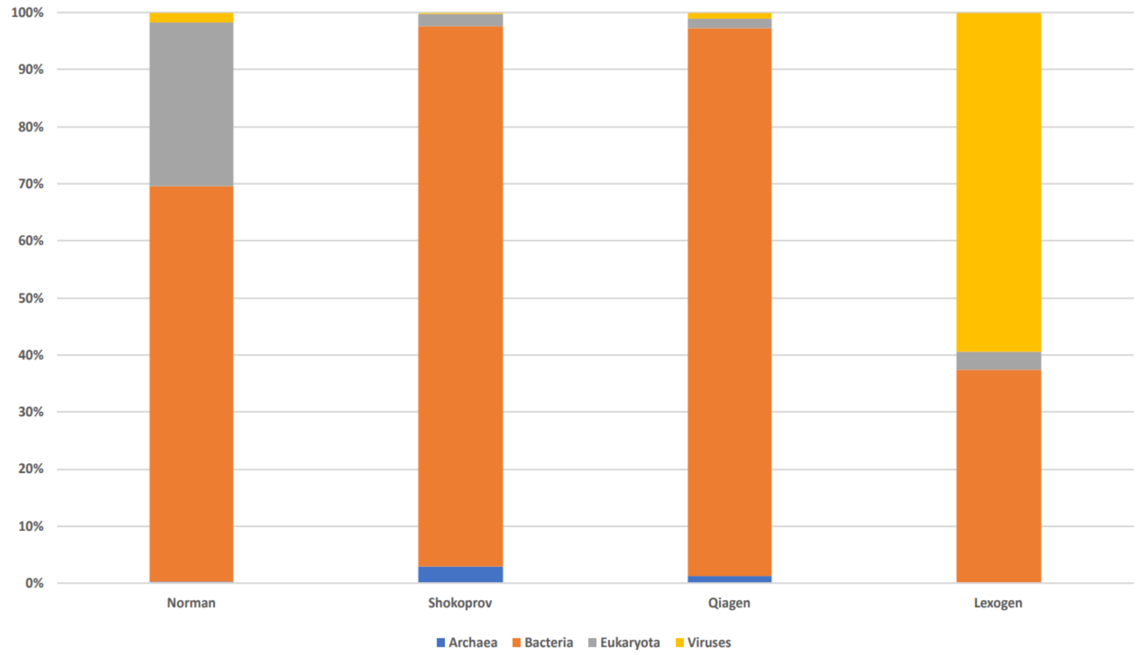

Figure S1. Optimization of urinary metagenome and virome protocols. (**A**) amplification of bacterial and fungal marker genes of urinary metagenome. (**B**) microbial roots of urinary metagenome in each donor urine sample using T3 protocol. (**C**) tested protocols for urinary virome. S1 (donor sample 1), S2 (donor sample 2), T1 (treatment 1), T2 (treatment 2), and T3 (treatment 3).
