# Supplementary material for: Characterization of the Urinary Metagenome and Virome in Healthy Children
Source: Biomedicines. 2022 Sep 27;10(10):2412. doi: 10.3390/biomedicines10102412 (PMC9599034; doi:10.3390/biomedicines10102412)
Supplement: Supplementary file 1 [file biomedicines-10-02412-s001.zip › Figure S2.pdf]

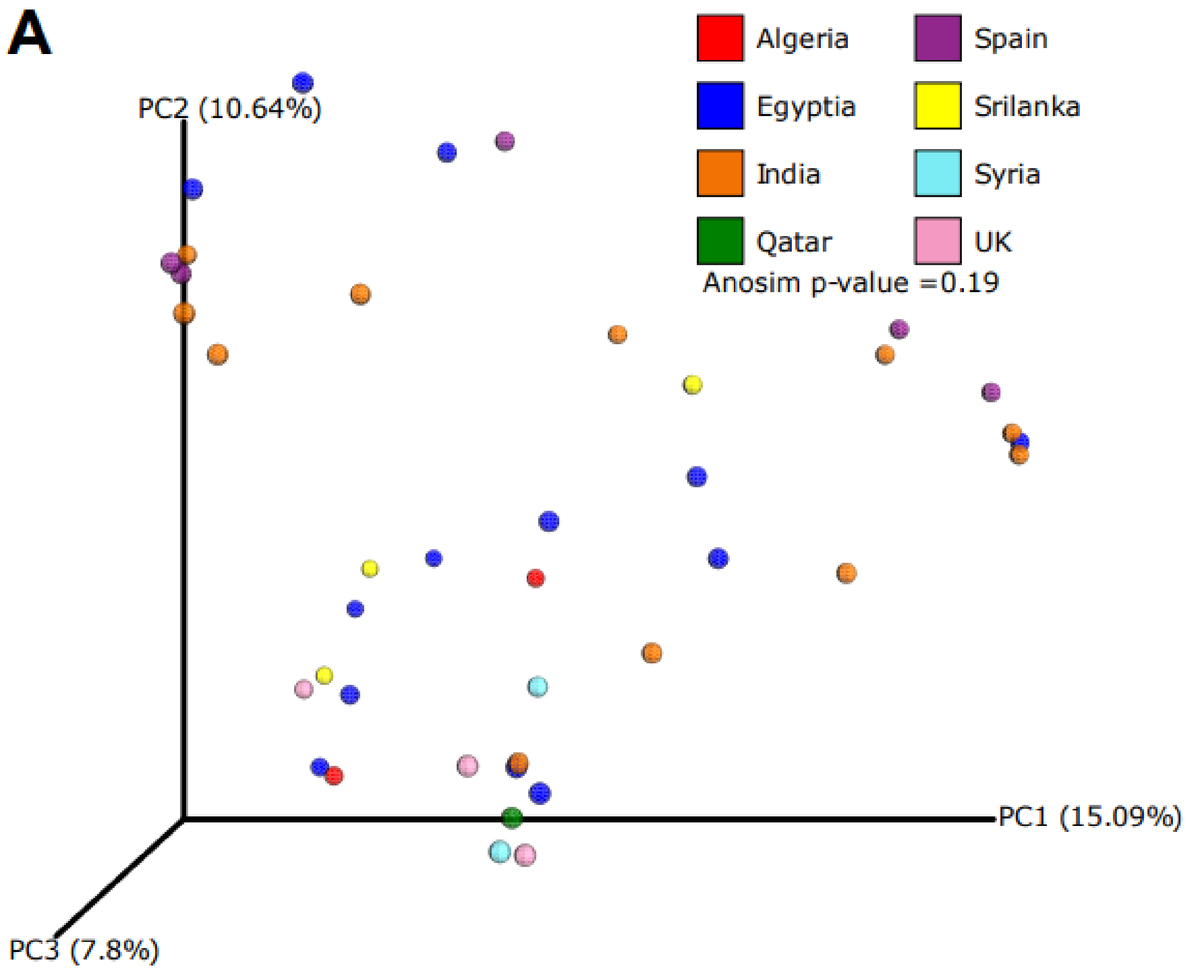
A
Algeria
Egyptia
India
Qatar
Spain
Srilanka
Syria
UK
Anosim p-value =0.19
PC2 (10.64%)
PC1 (15.09%)
PC3 (7.8%)

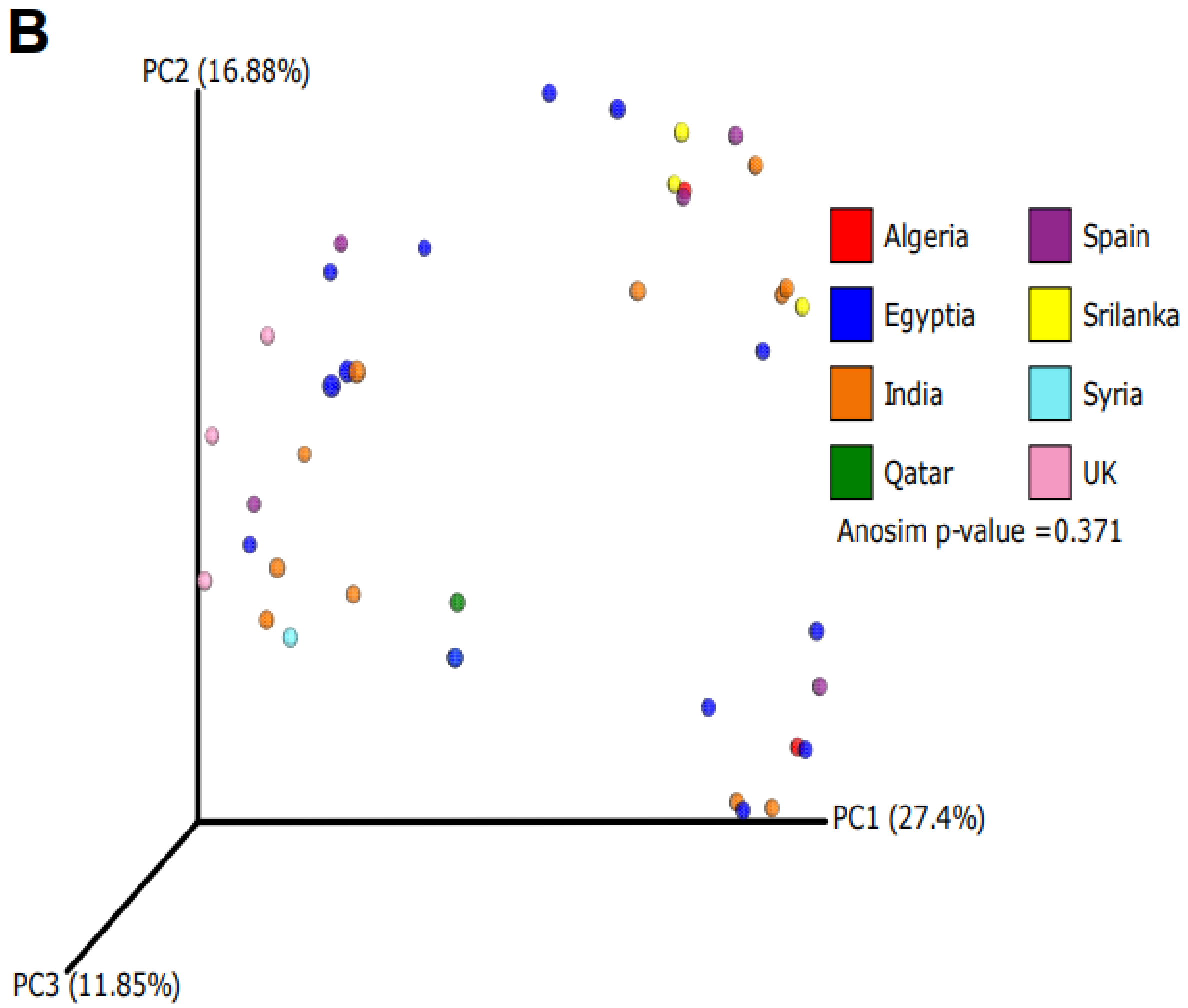
B
PC2 (16.88%)
PC1 (27.4%)
PC3 (11.85%)
Algeria
Egyptia
India
Qatar
Spain
Srilanka
Syria
UK
Anosim p-value =0.371

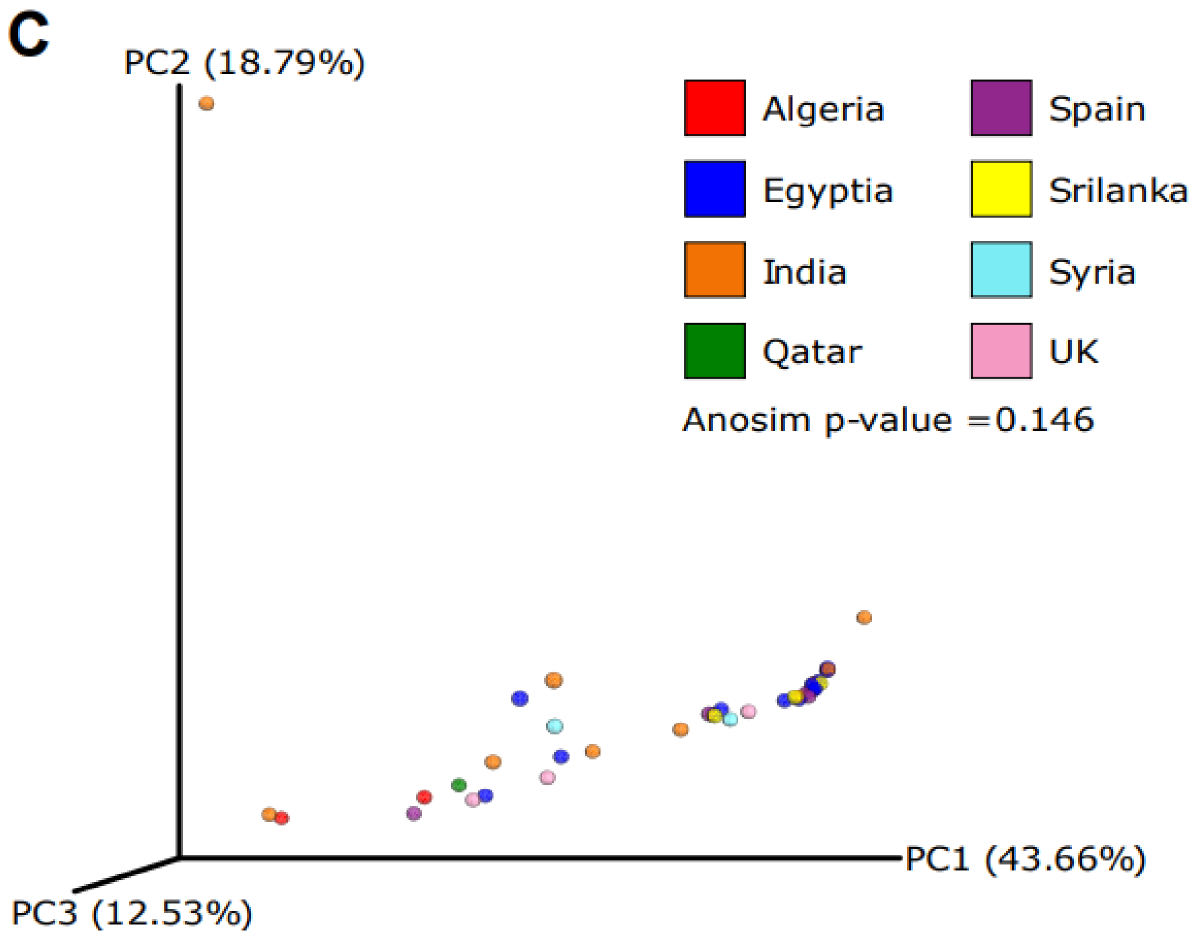

Figure S2. Beta diversity of the different nationalities of the healthy children. (**A**) beta diversity of urinary bacteriome. (**B**) beta diversity of urinary mycobiome. (**C**) beta diversity of urinary virome.
