# Supplementary material for: Characterization of the Urinary Metagenome and Virome in Healthy Children
Source: Biomedicines. 2022 Sep 27;10(10):2412. doi: 10.3390/biomedicines10102412 (PMC9599034; doi:10.3390/biomedicines10102412)
Supplement: Supplementary file 1 [file biomedicines-10-02412-s001.zip › Table S1.pdf]

Primer	Specific Primer sequence	Target Gene
NXT-16S_V3-V4_F	CCTACGGGNGGCWGCAG	16s rRNA
NXT-16S_V3-V4-R	GACTACHVGGGTATCTAATCC	
NXT-18S_F	GTACACACCGCCCGTC	18s rRNA
NXT-18S_R	TGATCCTTCTGCAGGTTCACC TAC	
NXT_ITS1_F	CTTGGTCATTTAGAGGAAGTA A	ITS2
NXT_ITS2_R	GCTGCGTTCTTCATCGATGC	

Table S1. Primers list of bacterial and fungal marker genes. 16srRNA (bacterial marker gene), 18srRNA and ITS2 (fungal marker genes).
